# High *SRD5A3* expression is correlated with promotion of proliferation and inhibition of apoptosis in B-cell non-Hodgkin lymphoma and suggests a poor prognosis

**DOI:** 10.1371/journal.pone.0323965

**Published:** 2025-05-21

**Authors:** Shanshan Wei, Jie Sun, Jiahao Wen, Jianing Yu, Yixuan Xuan, Jingyu Huang, Jie Yang, Jianfeng Zhang

**Affiliations:** 1 Department of Emergency, The Second Affiliated Hospital of Guangxi Medical University, Nanning, Guangxi, China; 2 Department of Hematological Oncology and Pediatric Oncology, Guangxi Medical University Cancer Hospital, Nanning, Guangxi, China; 3 Department of Imaging Medicine, Guigang City People’s Hospital, Guigang, Guangxi, China; 4 Department of general practice, The Second Affiliated Hospital of Guangxi Medical University, Nanning, Guangxi, China; 5 The Center for Clinical Medical Research, The Second Affiliated Hospital of Guangxi Medical University, Nanning, Guangxi, China; European Institute of Oncology, ITALY

## Abstract

**Background:**

Steroid 5α-reductase 3 (*SRD5A3*) is an important molecule involved in glycosylation and steroid hormone formation and is highly expressed in most tumors. However, The role of *SRD5A3* in B-cell non-Hodgkin lymphoma (B-NHL) and its mechanism are unknown.

**Methods:**

We used a multi-omics database to explore the expression and prognostic significance of *SRD5A3* in various tumors, including B-NHL. We established *SRD5A3* high- and low-expression B-NHL cell lines to test the effects of *SRD5A3* on cell proliferation and apoptosis in vitro, and to analyze the signaling pathways associated with the effects of *SRD5A3* on B-NHL.

**Results:**

We found that *SRD5A3* was highly expressed in most tumors, including B-NHL, and was more highly expressed in patients age ≥60 years, high levels of LDH, stage III-IV, non-GCB subtype, and extra-nodal invasion. Survival analysis showed that high *SRD5A3* expression predicted poorer overall survival (OS). Further experiments showed that *SRD5A3* high expression promoted B-NHL growth and attenuates apoptosis, conversely, *SRD5A3* low expression inhibited B-NHL growth and promoted apoptosis. Western blot assay showed *SRD5A3* promotes B-NHL cells growth by regulating the PI3K-AKT signaling pathway.

**Conclusions:**

Our findings suggest that *SRD5A3* exerts its oncogenic effects by regulating the PI3K-AKT pathway, may serve as a potential biomarker and therapeutic target for B-NHL, providing information for clinical decision-making.

## 1. Introduction

Non-Hodgkin’s lymphoma (NHL) is a heterogeneous disease caused by malignant transformation of lymphocytes, with 60–80% originating from B cells. Aggressive B-cell non-Hodgkin lymphoma (B-NHL) is the most common subtype of NHL, mainly comprising diffuse large B-cell lymphoma (DLBCL), Burkitt’s lymphoma (BL), and Mantle-cell lymphoma (MCL) [1–3], and is a clinically and genetically heterogeneous disease. Current treatments for B-NHL include rituximab + cyclophosphamide + vincristine + doxorubicin + prednisone (R-CHOP)-targeted chemotherapy, autologous stem cell transplantation (ASCT) and chimeric antigen receptor T cell (CAR-T) therapy. Despite significant advances in the treatment and management of B-NHL, approximately 30%-50% of B-NHL patients exhibit disease progression or relapse, the mechanisms of which are not fully understood [4,5]. One of the main reasons for this is resistance to chemotherapeutic agents. Resistance may be related to overexpression of specific proteins on the tumor cell membrane or within the tumor cells caused by genetic mutations [6]. Gene-targeted therapies have emerged as a potential strategy to overcome resistance to cancer chemotherapeutic agents.

According to data released by the Global Cancer Center, the incidence of lymphoma in China is about 6.68/100,000 people, with approximately 100,000 new cases each year, and the incidence of B-NHL in China is on the rise [7]. Risk stratification included the International Prognostic Index (IPI), age-adjusted International Prognostic Index (aaIPI), and National Comprehensive Cancer Network International Prognostic Index (NCCN-IPI) [8,9]. Differences in gene expression profiles (GEPs) and gene alterations can lead to significant differences in treatment responses and clinical courses [10]. With the advent and development of next-generation sequencing technologies, many studies have shown that genetic alterations, such as EZH2, MYD88, CD79B, and BCL2, are associated with the development of B-NHL [11–15]. The identification of new biomarkers may help refine risk stratification, inform treatment decisions, and predict treatment response and clinical outcomes.

Steroid 5 alpha-reductase 3(*SRD5A3*) is a protein-coding gene belonging to the steroid 5-alpha reductase family and is important for glycosylation metabolism and steroid hormone formation [16]. Steroid hormones are essential for mammalian stress response, immune system regulation, and reproduction. Glycosylation is an important post-translational modification that can alter the conformation and stability of proteins [17]. Aberrant glycosylation is closely associated with several pathological processes, including tumorigenesis and inflammatory responses [18]. Many studies have reported high expression of *SRD5A3* in liver, breast, endometrial, and prostate cancers, and its high expression is associated with poor prognosis [19–21], which is a cancer-promoting effect and is involved in the proliferation of cancer cells [16,22]. However, the clinical significance and mechanism of action of *SRD5A3* in B-NHL remain unclear.

In this study, in order to evaluate the prognostic factors of B-NHL, we analyzed the clinical data of B-NHL patients using a multi-omics database and analyzed the relationship between *SRD5A3* expression and clinicopathological characteristics and overall survival (OS) of B-NHL patients. We also investigated the role of *SRD5A3* in the development of B-NHL by in vitro functional assays and explored the potential mechanisms by which *SRD5A3* regulates cell proliferation and apoptosis.

## 2. Materials and methods

### 2.1 Bioinformatics analysis

We used B-NHL datasets from several different databases: (1)The pan-cancer dataset was downloaded from the UCSC database (https://xenabrowser.net/), and the expression data of *SRD5A3* gene were extracted, including 12,713 cancer samples and 10,505 normal samples; (2) *SRD5A3* transcript expression and survival information of 47 B-NHL patients from The Cancer Genome Atlas (TCGA; https://cancergenome.nih.gov/) database; (3) transcriptomic data from 337 normal donors obtained from the Genotype-Tissue Expression Database (GTEx; https://gtexportal.org); (4) *SRD5A3* transcript expression and survival information of 233 B-NHL patients, 55 B-NHL patients, 33 normal control samples,76 B-NHL patients, and 87 normal control samples from the Gene Expression Omnibus (GEO) database (https://www.ncbi.nlm.nih.gov/geo/; accession numbers GSE10846, GSE56315, and GSE83632). The public database data used in our study were publicly available online, and the experiments in this study had no ethical implications, so no additional ethical and moral statements were required.

### 2.2 Plasmid

For the shRNA plasmid, the lentiviral vector pHBLV-U6-MCS-CMV-ZsGreen-PGK-PURO was employed. A negative control (NC) virus, HBLV-ZsGreen-PURO NC, was designed with the following primers: sense strand: 5′-GATCCGTTCTCCGAACGTGTCACGTAATTCAAGAGATTACGTGACACGTTCGGAGAATTTTTTC-3′, antisense strand: 5′-AATTGAAAAAATTCTCCGAACGTGTCACGTAATCTCTTGAATTACGTGACACGTTCGGAGAACG-3′. The shRNA sequence targeting *SRD5A3* (HBLV-h-*SRD5A3* shRNA1-ZsGreen-PURO) was synthesized and cloned into the vector using the following primers: sense strand: 5′-GATCCGTGCATTCTTAGTGCTAGTATTctcgagAATACTAGCACTAAGAATGCATTTTTTG-3′, antisense strand: 5′-AATTCAAAAAATGCATTCTTAGTGCTAGTATTctcgagAATACTAGCACTAAGAATGCACG-3′. For the overexpression plasmid, the lentiviral vector pHBLV-CMV-MCS-EF1-ZsGreen-T2A-puro was utilized. The empty vector (HBLV-ZsGreen-PURO) served as the control. The *SRD5A3* overexpression construct (HBLV-h-*SRD5A3*-Null-ZsGreen-PURO) was generated using the following primers: sense primer: 5′-ctagaggatctatttccggtgaattcgccaccATGGCTCCCTGGGCGGAGGC-3′, antisense primer: 5′-agatccttactagtatcgatggatccTTAAAACAAAAATGGTAGGAAAG-3′.

### 2.3 Cell culture and transfection

The human B-cell lymphoma cell line SU-DHL-4 was purchased from Procell (Wuhan, China) and Raji was purchased from the Chinese Academy of Sciences (Shanghai, China). Both cell lines were cultured in RPMI-1640 medium containing 10% fetal bovine serum (FBS) and 1% antibiotics. To construct cell lines overexpressing or knocking down *SRD5A3*, SU-DHL-4 and Raji cells were infected with lentiviruses containing the indicated plasmids (HBLV-ZsGreen-PURO, HBLV-h-*SRD5A3*-Null-ZsGreen-PURO, HBLV-ZsGreen-PURO NC, and HBLV-h-*SRD5A3*-shRNA1-ZsGreen-PURO), respectively. Raji cells were knocked down *SRD5A3* to obtain sh-*SRD5A3* cells, and SU-DHL-4 cells were overexpressed *SRD5A3* to obtain oe-*SRD5A3* cells, their controls were sh-NC cells and oe-NC cells, respectively.

### 2.4 Quantitative Real-time PCR

TRIzol reagent (Invitrogen) was used to extract total RNA, which was reverse transcribed into cDNA. Real-time fluorescence quantitative PCR was performed using one-step Hieff qPCR SYBR Green Master Mix (No Rox) (Yeasen, China) according to the manufacturer's instructions. The relative levels of *SRD5A3* cDNA were calculated as 2-ΔΔCt and normalized to the human GAPDH gene. The upstream primer for *SRD5A3* was 5’-TGGCTGCACAGCTTACGAAG- 3’ and the downstream primer was 5-TCAGCACAGTTAGGCCAACAA-3’; for GAPDH, the upstream primer was 5’-GCACCGTCAAGGCTGAGAAC-3’ and the downstream primer was 5’- TGGTGAAGACGCCAGTGGA- 3’.

### 2.5 Cell proliferation assay

The CCK-8 assay was used to determine cell proliferation. *SRD5A3* overexpression cells oe-*SRD5A3*, control cells oe-NC, *SRD5A3* knockdown cells sh-*SRD5A3*, and control sh-NC cells were inoculated in 96-well plates at a density of 1 × 10^5^ cells/mL, and three replicate wells were prepared for each group of cells. At 0h, 24h, 48h and 72h, 10 μL of CCK-8 working solution was added and incubated for 3h away from light, and he absorbance was measured at 450 nm (OD450). Growth curves for each group of cells were plotted.

### 2.6 Apoptosis assay

The apoptosis rate was determined using an Annexin V-APC/7-AAD Apoptosis Kit (MULTISCIENCES, China). The oe-*SRD5A3* and oe-NC cells and sh-*SRD5A3* and sh-NC cells with good growth status and more than 80% cell fusion were taken and stained with membrane-bound proteins V-APC and 7-AAD according to the instructions, so that the liquid was mixed well, and then incubated for 15 minutes at room temperature away from light, and apoptosis was detected by using flow cytometer in 1 h. The cells were then incubated for 15 min at room temperature in the dark, and apoptosis was detected using flow cytometry. The experiments were performed in 3 biological replicates.

### 2.7 Western blot

Cells with a growth density of 80% or higher were collected, washed twice with pre-cooled PBS, added to an appropriate amount of cell lysate (RIPA Total Protein Lysate: PMSF = 100:1, prepared 5 min before use), mixed well, and lysed on ice for 30 min. The resulting cell lysates were centrifuged at 12,000 rpm for 15 minutes at 4°C. The supernatant was aspirated into a new pre-cooled 1.5 ml EP tube, and the protein concentration was detected and recorded on a UV spectrophotometer. Proteins were separated by SDS-PAGE and electrotransferred onto polyvinylidene fluoride (PVDF) membranes. After 1 h of closure at room temperature, the membrane was incubated with the primary antibody at 4°C overnight, followed by incubation with the secondary antibody for 1–2 hours at room temperature. Blots were visualized using the Odyssey gel image system and analyzed using the ImageJ software. Primary antibodies against GAPDH, *SRD5A3*, BCL-2, PCNA, N-cadherin, Vimentin, PI3K, p-PI3K, AKT and p-AKT were purchased from Proteintech (Wuhan, China).

### 2.8 Statistical analysis

R 4.3.1, SPSS 26.0, and GraphPad Prism 9 software were used to analyze the data, and graphs, respectively. Data are expressed as the mean ± standard deviation (X ± SD). Comparison of the means of the two samples was performed using the t-test; univariate survival analysis was performed using the Kaplan-Meier method and tested by the log-rank method, and multifactorial survival analysis was analyzed by Cox regression. Statistical significance was set at p < 0.05.

## 3. Results

### 3.1 SRD5A3 is highly expressed in most tumors including B-NHL and suggests a poor prognosis

**Fig 1:**
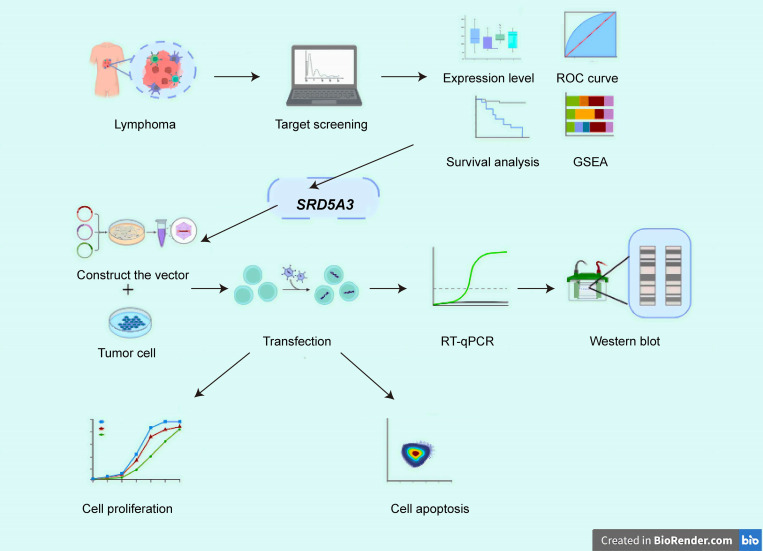
Workflow chart.

**Fig 2:**
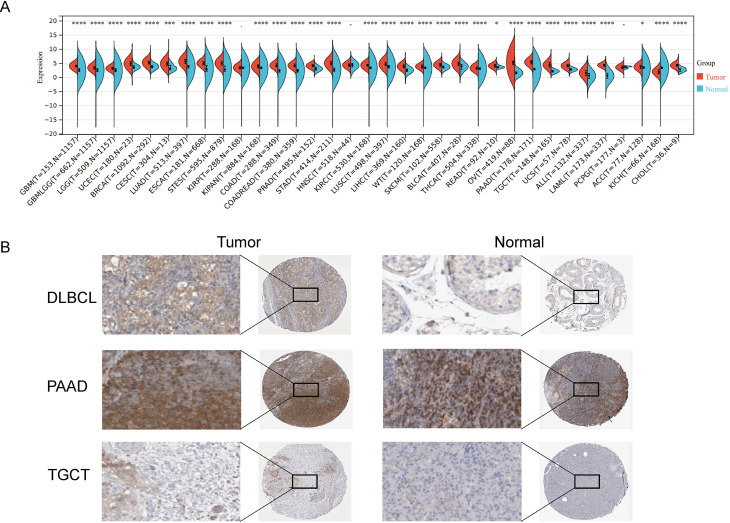
Expression of *SRD5A3* in pan-cancer. **A.** Expression of *SRD5A3* in pan-cancer and normal tissues. B. Immunohistochemistry of *SRD5A3* in B-NHL, PAAD, TGCT and normal tissues. The significance is marked as p < 0.05 *; p < 0.01 **; p < 0.001 ***; p < 0.0001 ****. Abbreviation: GBM, Glioblastoma; HNSC, GBMLGG, Glioma; LGG, Lower Grade Glioma; UCEC, Endometrioid Cancer; BRCA,Breast Cancer; CESC, Cervical Cancer; LUAD, Lung Adenocarcinoma; ESCA, Esophageal carcinoma; STES, Stomach and Esophageal carcinoma; KIRP,Kidney Papillary Cell Carcinoma; KIPAN, Pan-kidney cohort (KICH+KIRC+KIRP); COAD, Colon Cancer; COADREAD, Colon adenocarcinoma/Rectum adenocarcinoma Esophageal carcinoma; PRAD, Prostate Cancer; STAD,Stomach Cancer; HNSC, Head and Neck squamous cell carcinoma; KIRC, Kidney renal clear cell carcinoma; LUSC, Lung Squamous Cell Carcinoma; LIHC, Liver hepatocellular carcinoma; WT, Wilms Tumor; SKCM, Melanoma; BLCA, Bladder Urothelial Carcinoma; THCA,Thyroid Cancer; READ, Rectal Cancer; OV, Ovarian Cancer; PAAD, Pancreatic Cancer; TGCT, Testicular Cancer; UCS, Uterine Carcinosarcoma; ALL, Acute Lymphoblastic Leukemia; LAML, Acute Myeloid Leukemia; PCPG,Pheochromocytoma & Paraganglioma; ACC, Adrenocortical Cancer; KICH,Kidney Chromophobe; CHOL, Bile Duct Cancer.

**Fig 3:**
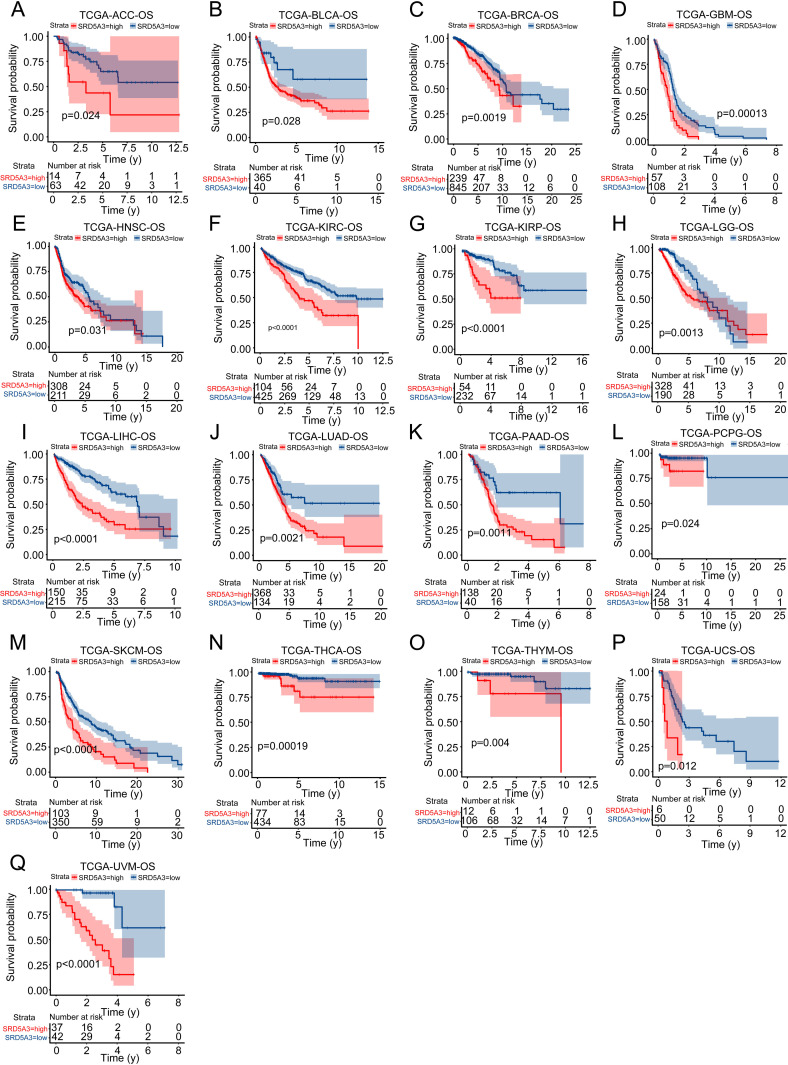
Survival analysis of *SRD5A3* in pan-cancer. P < 0.05 indicates statistical significance.

The workflow diagram is shown in Fig 1. To determine the expression of *SRD5A3* in pan-cancer and its relationship with prognosis, we collected *SRD5A3* transcriptional expression and survival information of various tumors and normal control samples from TCGA and GTEx databases and analyzed the expression of *SRD5A3* in various tumor and normal control tissues using Sangerbox software (Fig 2A). We used The Human Protein Atlas (HPA) website to analyze the immunohistochemistry of *SRD5A3* in tumor and normal tissues and found that the expression of *SRD5A3* was higher in lymphoma, pancreatic cancer, and testicular cancer than in normal tissues (Fig 2B). We used the R package to perform pan-cancer survival analysis of *SRD5A3* (Fig 3). The results showed that *SRD5A3* was highly expressed in most tumors compared to normal tissues, and patients with *SRD5A3* high expression had shorter overall survival.

### 3.2 SRD5A3 expression is associated with adverse clinicopathologic features in B-NHL

**Fig 4:**
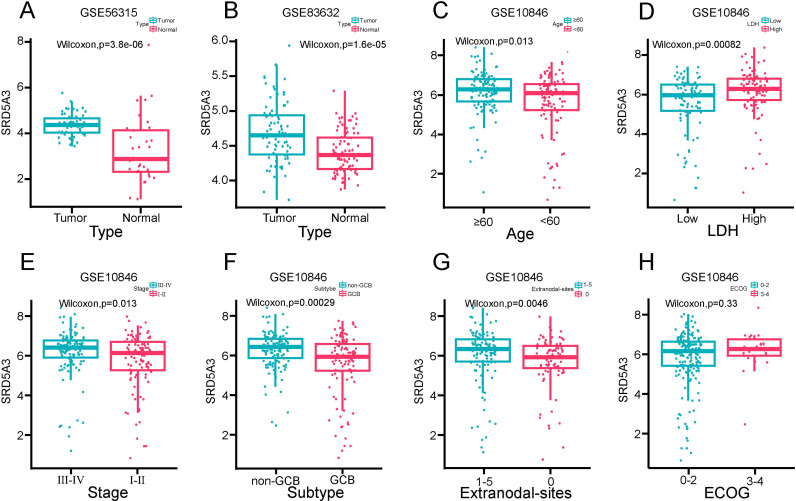
Expression of *SRD5A3* in various clinicopathologic features in B-NHL. A. *SRD5A3* expression in B-NHL and normal tissues in the GSE56315 dataset. B. *SRD5A3* expression in B-NHL and normal tissues in the GSE83632 dataset. C. *SRD5A3* expression in different age groups. D. *SRD5A3* expression in different LDH levels. E. *SRD5A3* expression in different clinical stages. F. *SRD5A3* expression in different subtype. G. *SRD5A3* expression in different numbers of Extranodal invasion site. H. *SRD5A3* expression in different ECOG scores. P < 0.05 indicates statistical significance.

To understand the expression of *SRD5A3* in B-NHL and normal tissues, we analyzed the expression of *SRD5A3* in 55 B-NHL patients and 33 normal control samples in the GSE56315 dataset, as well as analyzed the expression of *SRD5A3* in 76 B-NHL patients and 87 normal control samples in the GSE83632 dataset. The results showed that the expression of *SRD5A3* was significantly higher in B-NHL tissues than in normal tissues (Fig 4A-B). We also analyzed the differential expression of *SRD5A3* in different clinicopathological subgroups of the GSE10846 dataset, the same treatment regimen was used for all of these sample. The results showed that *SRD5A3* expression was higher in patients with ≥60 years old, high LDH levels, stage III-IV, non-GCB subtype, and extranodal invasion (Fig 4C-G), whereas there were no significant differences in ECOG score subgroups (Fig 4H).

### 3.3 Construction of a prognostic model containing SRD5A3

**Fig 5:**
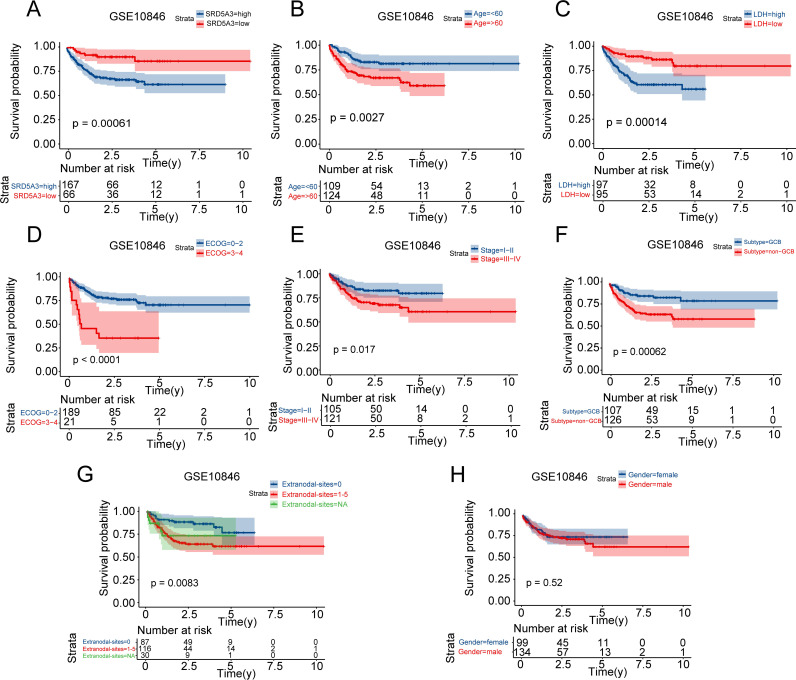
Univariate COX regression analysis of different clinicopathologic features on OS in B-NHL. A. Effect of *SRD5A3* expression on OS. B. Effect of age on OS. C. Effect of LDH on OS. D. Effect of ECOG score on OS. E. Effect of clinical stage on OS. F. Effect of subtype on OS. G. Effect of the number of extranodal invasion sites on OS. H. Effects of gender on OS. P < 0.05 indicates statistical significance.

To investigate whether *SRD5A3* could serve as a prognostic marker for B-NHL, we analyzed the effects of different clinicopathological features on OS in the GSE10846 dataset using univariate COX regression. the same treatment regimen was used for all of these sample. The results showed that patients in the group with low *SRD5A3* expression, age < 60 years, low LDH level, low ECOG score, low clinical stage, GCB subtype, and patients without extranodal invasion had a longer overall survival (Fig 5A-G), whereas sex had no significant effect on prognosis (Fig 5H).

**Fig 6:**
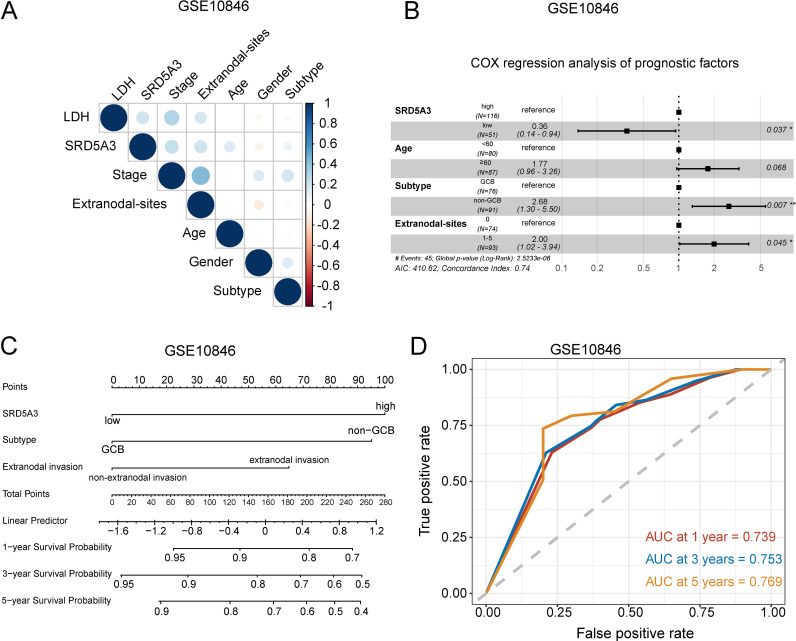
Multivariate COX regression analyses and construction of prognostic prediction model. A. Correlation analysis between *SRD5A3* and different clinicopathologic features. B. Multivariate COX regression analysis of OS. C. Nomogram for prognostic modeling. D. ROC curve of the prediction model. The significance is marked as p < 0.05 *; p < 0.01 **; p < 0.001 ***; p < 0.0001 ****; p < 0.05 indicates statistical significance.

Next, we performed a correlation analysis between *SRD5A3* and these clinicopathological features, which showed that there were correlations between *SRD5A3* and LDH and stage, whereas the correlations were not significant for age, sex, ECOG score, subtype, and extranodal invasion (Fig 6A). This shows that there is covariance between *SRD5A3* and LDH and stage. We combined the results of expression analysis, univariate COX regression analysis and correlation analysis, we included *SRD5A3*, age, subtype, and extranodal invasion in multivariate COX regression survival analysis and found that *SRD5A3*, subtype, and extranodal invasion were independent prognostic factors for OS in B-NHL patients, the C-index of nomogram was 0.72*5* (Fig 6B-C). These factors were combined to establish a prognostic prediction model for B-NHL, and the ROC curve showed that this prognostic model had a moderate ability to predict the 1-year, 3-years and 5-years survival rates of B-NHL patients (Fig 6D).

### 3.4 Signaling pathways associated with SRD5A3

**Fig 7:**
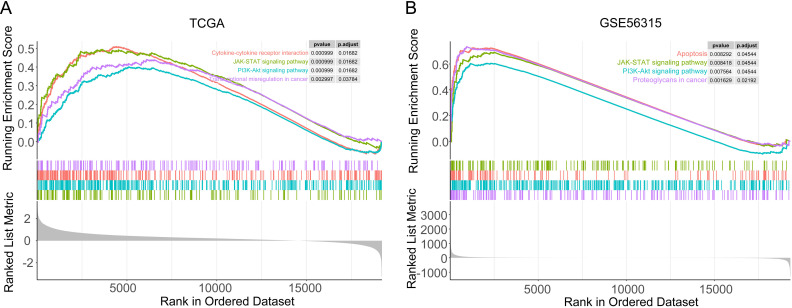
Gene Set Enrichment Analysis. A. Pathway enrichment analysis of the TCGA dataset. B. Pathway enrichment analysis of the GSE56315 dataset. P < 0.05 indicates statistical significance.

To explore the relevant signaling pathways for *SRD5A3* action, we performed GSEA pathway enrichment analysis on TCGA and GSE56315 datasets. The results showed that the differentially expressed genes were mainly enriched in signaling pathways such as apoptosis, JAK-STAT, PI3K-AKT, and transcription factor dysregulation in cancer (Fig 7A-B). These results suggest that *SRD5A3* promotes tumor development through these pathways.

### 3.5 Lentiviral transfection to construct SRD5A3 overexpression and suppressed expression cell lines

**Fig 8:**
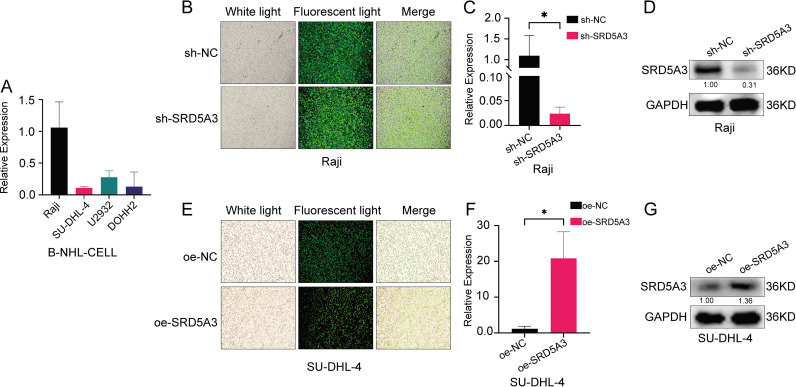
Construction of cell models with *SRD5A3* suppressed expression and overexpression. A.*SRD5A3* expression in B cell lymphoma cells. B. Fluorescence after *SRD5A3* suppressed expression. C. *SRD5A3* cDNA levels after *SRD5A3* suppressed expression. D. *SRD5A3* protein levels after *SRD5A3* suppressed expression. E. Fluorescence after *SRD5A3* overexpression. F. *SRD5A3* cDNA levels after *SRD5A3* overexpression. G. *SRD5A3* protein levels after *SRD5A3* overexpression. The significance is marked as p < 0.05 *; p < 0.05 indicates statistical significance.

To alter *SRD5A3* expression in vitro, we performed RT-qPCR analysis on several B-NHL cells, knocked down the highest *SRD5A3*-expressed Raji cells, and overexpressed the lowest *SRD5A3*-expressed SU-DHL-4 cells (Fig 8A). Lentiviral constructs carrying oe-*SRD5A3*, sh-*SRD5A3*, and the corresponding empty vectors were transfected into SU-DHL-4 and Raji cells to overexpress or knockdown *SRD5A3*. Cellular green fluorescent protein (GFR) expression was observed under an inverted fluorescence phase-contrast microscope (Fig 8B,8E), and *SRD5A3* cDNA and protein levels were detected by RT-qPCR and western blotting. The results showed that sh-NC and oe-NC did not alter *SRD5A3* expression. The *SRD5A3* cDNA level was significantly decreased in sh-*SRD5A3* and increased in oe-*SRD5A3* (Fig 8C,8F); whereas *SRD5A3* protein levels were significantly decreased in sh-*SRD5A3* and increased in oe-*SRD5A3*(Fig 8D,8G).

### 3.6 Upregulation of SRD5A3 promotes proliferation of B-cell non-Hodgkin lymphoma cells

**Fig 9:**
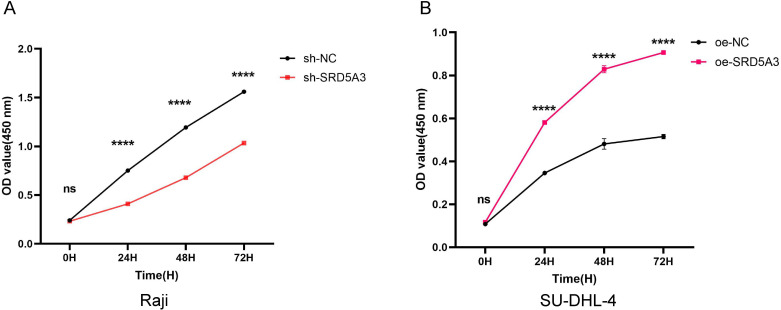
Effect of *SRD5A3* on the proliferation in B-cell non-Hodgkin lymphoma. A. Inhibition of *SRD5A3* expression suppresses proliferation. B. Overexpression of *SRD5A3* promotes proliferation. The significance is marked as p < 0.05 *; p < 0.01 **; p < 0.001 ***; p < 0.0001 ****; p < 0.05 indicates statistical significance.

To investigate whether *SRD5A3* affects the proliferation of B-NHL cells, the CCK-8 assay was used to detect the proliferation of cells in the oe-*SRD5A3* and oe-NC groups and the sh-*SRD5A3* and sh-NC groups. Cell growth curves were plotted based on the OD results. We found that knockdown of *SRD5A3* (sh-*SRD5A3*) resulted in significantly lower proliferative activity than that of control cells (sh-NC) (Fig 9A). In contrast, the proliferative activity of overexpressing *SRD5A3* (oe-*SRD5A3*) was significantly higher than that of control cells (oe-NC) (Fig 9B). These data suggest that the upregulation of *SRD5A3* promotes proliferation in B-NHL cell, conversely, the downregulation of *SRD5A3* inhibits proliferation in B-NHL cells.

### 3.7 Upregulation of SRD5A3 inhibits apoptosis of B-cell non-Hodgkin lymphoma cells

**Fig 10:**
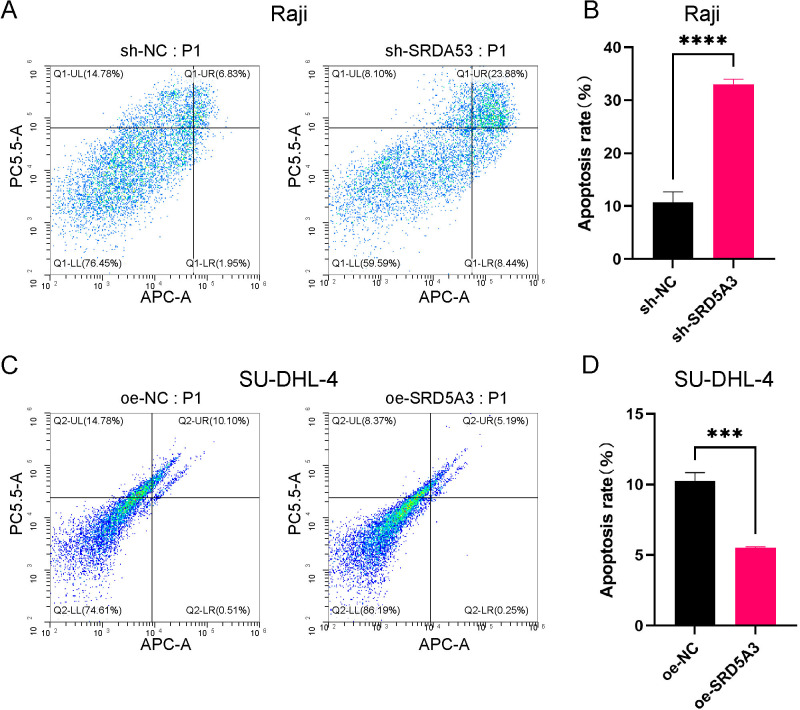
Effect of *SRD5A3* on apoptosis in B-cell non-Hodgkin lymphoma. A. Inhibition of SRD5A3 expression promotes apoptosis. B. Overexpression of *SRD5A3* suppresses apoptosis. The significance is marked as p < 0.05 *; p < 0.01 **; p < 0.001 ***; p < 0.0001 ****; p < 0.05 indicates statistical significance.

To investigate whether *SRD5A3* affects cell proliferation by regulating apoptosis, we performed flow cytometry analysis of cells in the sh-*SRD5A3* and sh-NC and oe-*SRD5A3* and oe-NC groups. The results showed that the apoptosis rate was 33.05 ± 0.93% in the sh-*SRD5A3* group and 10.69 ± 2% in the sh-NC group, and the apoptosis level in the sh-*SRD5A3* group was significantly higher than that in the sh-NC group (Fig 10A); the apoptosis rate in the oe-*SRD5A3* group was 5.5 ± 0.09%, and the oe-NC group cell apoptosis rate was 10.25 ± 0.59%, and the apoptosis level in the oe-NC group was significantly higher than that in the oe-*SRD5A3* group (Fig 10B). These data indicate that the upregulation of *SRD5A3* attenuated apoptosis in B-NHL cell, conversely, the downregulation of *SRD5A3* promotes apoptosis in B-NHL cells.

### 3.8 Mechanism of SRD5A3 regulation of cell proliferation and apoptosis

**Fig 11:**
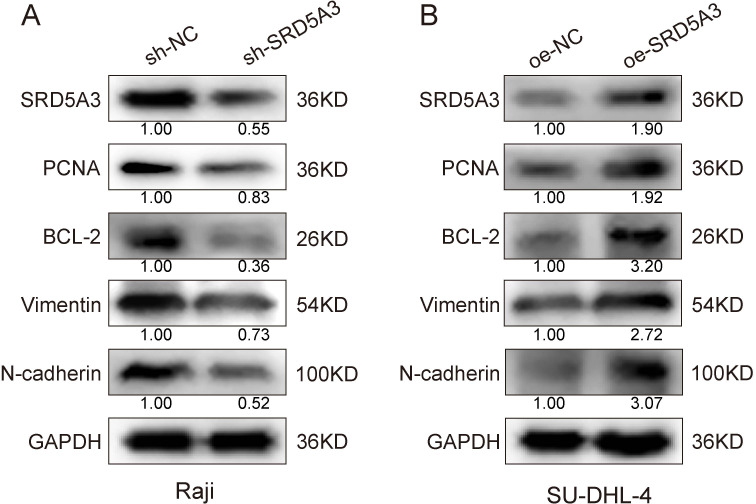
Mechanism of *SRD5A3* regulates B-NHL cell proliferation and apoptosis.

**Fig 12:**
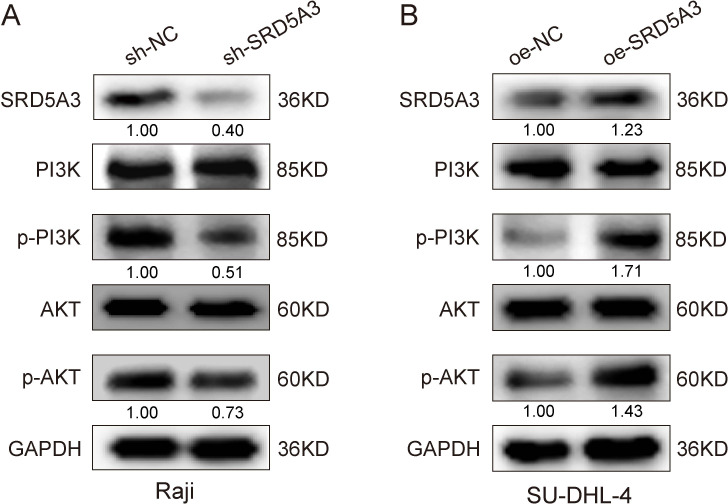
Signaling pathways associated with the function of *SRD5A3.*

**Fig 13:**
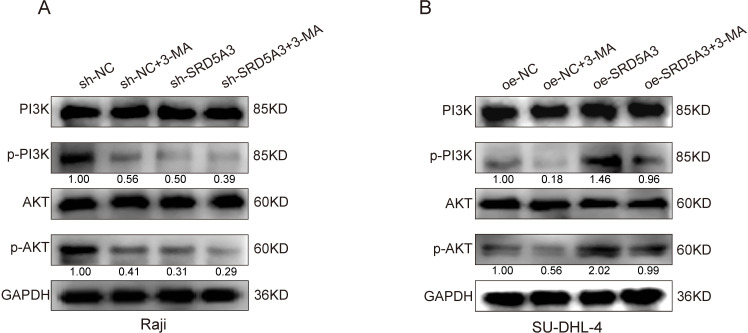
Effects of PI3K pathway inhibitors on PI3K-AKT signaling proteins in SRD5A3 knockdown and overexpression cells.

To investigate the molecular mechanism by which *SRD5A3* regulates cell growth, we used protein blotting to detect the expression of PCNA proliferative protein, Bcl-2 anti-apoptotic protein, N-cadherin and Vimentin adhesion protein. We found that *SRD5A3* overexpression increased the expression of PCNA, BCL-2, N-cadherin, and Vimentin. Conversely, *SRD5A3* knockdown decreased their expression (Fig 11). It can be seen that up-regulation of *SRD5A3* increased the expression of PCNA, BCL-2, N-cadherin and Vimentin, resulting in enhanced cell proliferation and migration and attenuated apoptosis. Moreover, we verified the changes in PI3K-AKT signaling by Western blot experiments, which showed that SRD5A3 overexpression increased the levels of p-PI3K and p-AKT signaling proteins, and vice versa, SRD5A3 low expression inhibited the levels of p-PI3K and p-AKT signaling proteins (Fig 12). To further confirm that *SRD5A3* affects B-NHL development by regulating the PI3K-AKT signaling pathway, we treated *SRD5A3* knockdown and control cells with the PI3K inhibitor 3-MA drug, followed by Western blot experiments. The results showed that in Raji cells, p-PI3K and p-AKT protein levels were highest in the sh-NC group, intermediate in the sh-NC + 3-MA and sh-*SRD5A3* groups, and lowest in the sh-*SRD5A3*+3-MA group (Fig 13A). These data suggest that *SRD5A3* knockdown reduces p-PI3K and p-AKT protein levels and that 3-MA drugs further enhance this reduction. Similarly, we treated *SRD5A3* overexpression group and control cells with 3-MA drugs and performed Western blot experiments. The results showed that in SU-DHL-4 cells, the cells in the oe-*SRD5A3* group had the highest levels of p-PI3K and p-AKT proteins, the oe-NC group and the oe-SRD5A3+3-MA group had intermediate levels of p-PI3K and p-AKT proteins, and the oe-NC + 3-MA group had the lowest levels of the proteins (Fig 13B). These data suggest that *SRD5A3* overexpression enhances p-PI3K and p-AKT protein levels, and that 3-MA reverses the enhancing effect of *SRD5A3* overexpression on p-PI3K and p-AKT protein levels. These experimental data further confirmed that *SRD5A3* acts through regulating the PI3K-AKT signaling pathway.

## 4. Discussion

*SRD5A3* is a member of the SRD5A family and is a protein-coding gene, which are located on chromosome 4 of the human genome and is approximately 36 kDa in length. It is an important molecule for glycosylation metabolism and steroid hormone formation [16]. Steroid hormones are essential for the mammalian stress response, immunomodulation, and reproduction. Steroids, testosterone, and progesterone are catalytically produced by steroid 5α-reductase (SRD5As) and are important in a variety of physiological and pathological processes [23]. Glycosylation is a very important form of post-translational modification of proteins, which directly interacts with the surrounding environment or indirectly alters the conformation and stability of the protein [17]. Glycosylated proteins are involved in a variety of biological processes, and aberrant glycosylation is closely associated with many pathological processes such as tumorigenesis and inflammatory responses [18]. Glycosylation modifications affect tumor cell migration and invasion not only through the connection between the extracellular matrix and transmembrane proteins but also by modulating metastasis-related molecular signaling pathways. For example, E-cadherin, integrins, and mucins can alter the structure and function of glycoproteins, leading to abnormal glycosylation and promotion of tumor metastasis [24]. Pu C et al. showed that abnormal glycosylation promotes the development of endometrial and breast cancer [24,25].

Non-Hodgkin’s lymphoma (NHL) is the most common hematologic malignancy arising from lymphoid tissue, with approximately 85% originating from B lymphocytes [26]. B-NHL is characterized by heterogeneity in clinical presentation and course, ranging from asymptomatic and inert to highly aggressive [27]. Chemoimmunotherapy remains the first-line standard of care for aggressive B-NHL. However, a study by Frontzek F et al. found that high-dose chemotherapy combined with autologous hematopoietic stem cell transplantation did not improve outcomes in high-risk aggressive B-cell lymphomas patients, but instead showed significant toxicity [28]. Therefore, the search for new targets to improve treatment and outcomes in B-NHL patients remains urgent. Further research on B-NHL-related genes will help to improve our understanding of the pathogenesis of the disease, thereby improving the treatment and survival of these patients. Our study found that *SRD5A3* was highly expressed in most tumors, such as B-NHL, breast, liver, colorectal, and ovarian cancer, compared to normal control tissues, and that patients with *SRD5A3* high expression had a poor OS. Studies have shown that *SRD5A3* expression is usually higher in a variety of tumor tissues than in non-tumor tissues. For example, *SRD5A3* is overexpressed in hepatocellular carcinoma, breast carcinoma, endometrial carcinoma, and prostate carcinoma, and patients with *SRD5A3* high expression suggest a poor prognosis [19–21]. We also explored the relationship between *SRD5A3* and the clinicopathological features and prognosis of the B-NHL dataset GSE10846 and established a prognostic prediction model that included *SRD5A3*. We found that *SRD5A3* expression was higher in patients with≥ 60 years old, high levels of LDH, stage III-IV, non-GCB subtype and extranodal invasion, while there were no significant differences in ECOG score subgroups. Survival analysis revealed longer OS in patients with low expression of *SRD5A3*, < 60 years old, low LDH levels, low ECOG scores, low clinical stage, GCB subtype and without extranodal invasion, whereas sex had no significant effect on prognosis. That is in agreement with the findings of Bardakci M [29–33] et al. Subsequently, we performed a multivariate COX regression analysis, which showed that *SRD5A3*, subtype and extranodal invasion were independent prognostic factors for OS in B-NHL patients. The C-index of nomogram was 0.725, indicating that the prognostic model had moderate predictive power. The results of the ROC curves showed that the prognostic model had a moderate ability to predict 1-,3- and 5-years survival in B-NHL patients.

We speculate that *SRD5A3* plays an important role in the pathogenesis of B-NHL and provide preliminary evidence supporting *SRD5A3* as a potential therapeutic target and prognostic marker. In SU-DHL-4 cells, *SRD5A3* overexpression promoted proliferation and attenuated apoptosis, whereas in Raji cells, *SRD5A3* knockdown inhibited proliferation and enhanced apoptosis. This suggests that *SRD5A3* is pro-carcinogenic and involved in the proliferation of cancer cells, which is consistent with previous findings in other tumors [16,22]. A study by Mai Q et al. used hepatocellular carcinoma cells with knocked down *SRD5A3* to de-inoculate the subcutaneous tissue of nude mice, and found that downregulation of *SRD5A3* inhibited hepatocellular carcinoma growth [16]. In our study, we found that upregulation of *SRD5A3* increased BCL-2 anti-apoptotic proteins, PCNA proliferative proteins, and N-cadherin and Vimentin proteins, conversely, downregulation of *SRD5A3* suppressed these protein levels.

We performed GSEA pathway enrichment analysis on the differentially expressed genes and found that they were mainly enriched in signaling pathways such as JAK-STAT, PI3K-AKT, apoptosis, and transcription factor dysregulation in cancer. Studies have reported mutations that activate JAK or other upstream oncogenes, which, in turn, activate STAT, leading to tumorigenesis [34]. Song et al. found that STAT3 is constitutively activated in tumors such as DLBCL, head and neck cancer, and colorectal cancer [35–37]. Many studies have reported that the PI3K-AKT-mTOR pathway is active in various tumors, and related drugs such as PI3K, AKT, and mTOR inhibitors have been used in the clinical treatment of DLBCL [38–40]. In this study, we verified how *SRD5A3* regulates PI3K-ATK signaling by Western blot assay, The results showed that downregulation of *SRD5A3* inhibited p-PI3K and p-AKT protein levels, and PI3K inhibitors further enhanced the inhibitory effect of *SRD5A3* knockdown on p-PI3K and p-AKT protein levels. In contrast, upregulation of *SRD5A3* enhanced p-PI3K and p-AKT protein levels, and PI3K inhibitors reversed the enhancement of p-PI3K and p-AKT protein levels by *SRD5A3* overexpression. These results further confirm that *SRD5A3* acts by regulating the PI3K-AKT signaling pathway.

## 5. Conclusion

In summary, we found that *SRD5A3* is highly expressed in most tumors, including B-NHL, and may play a role in the development of B-NHL, suggesting poor prognosis. *SRD5A3* promotes B-NHL cell proliferation and attenuates cancer cell apoptosis by regulating the PI3K-AKT signaling pathway. Our data suggest that *SRD5A3* functions as an oncogene, which may serve as a potential prognostic biomarker and therapeutic target for B-NHL.

However, the shortcoming of this study is that we did not further validate the effect of *SRD5A3* expression level on the growth of B-NHL by in vivo nude mice subcutaneous tumor formation experiments, which will be explored in the next step.

## Supporting information

S1 File The original uncropped and unadjusted Western blot images. (XLSX)

S2 File The original plotting data. (PDF)

## Abbreviations

*SRD5A3*: Steroid 5 alpha-reductase 3
B-NHL: B-cell non-Hodgkin lymphoma
LDH: lactate dehydrogenase
OS: overall survival
DLBCL: diffuse large B-cell lymphoma
BL: Burkitt’s lymphoma
MCL: Mantle-cell lymphoma
ASCT: autologous stem cell transplantation
CAR-T: chimeric antigen receptor T cell
IPI: International Prognostic Index
aaIPI: age adjusted International Prognostic Index
NCCN-IPI: NCCN International Prognostic Index
EZH2: enhancer of zeste homolog
MYD88: myeloid differentiation factor 88
CD79B: B-cell antigen receptor complex-associated protein beta chain
BCL-2: B-cell lymphoma-2
TCGA: The Cancer Genome Atlas
GTEx: Genotype-Tissue Expression
GEO: Gene Expression Omnibus
ECOG: Eastern Cooperative Oncology Group
GSEA: Gene Set Enrichment Analysis
RT-qPCR: Quantitative Real-time PCR.
