# Supplementary figures and images for: High SRD5A3 expression is correlated with promotion of proliferation and inhibition of apoptosis in B-cell non-Hodgkin lymphoma and suggests a poor prognosis
Source: PLoS One. 2025 May 21;20(5):e0323965. doi: 10.1371/journal.pone.0323965 (PMC12094769; doi:10.1371/journal.pone.0323965)

Fig8 D

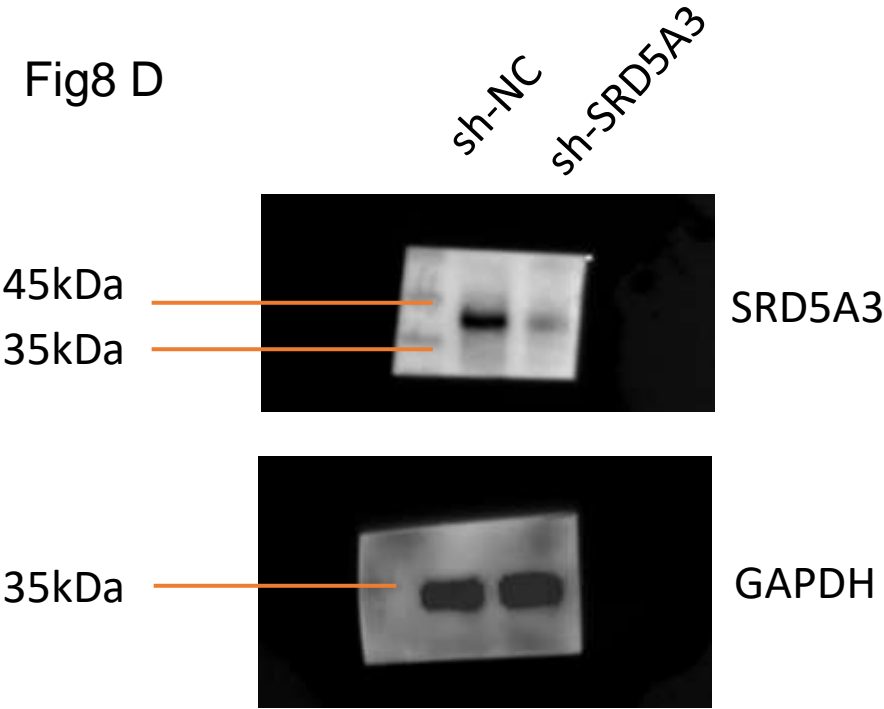

Fig8 G

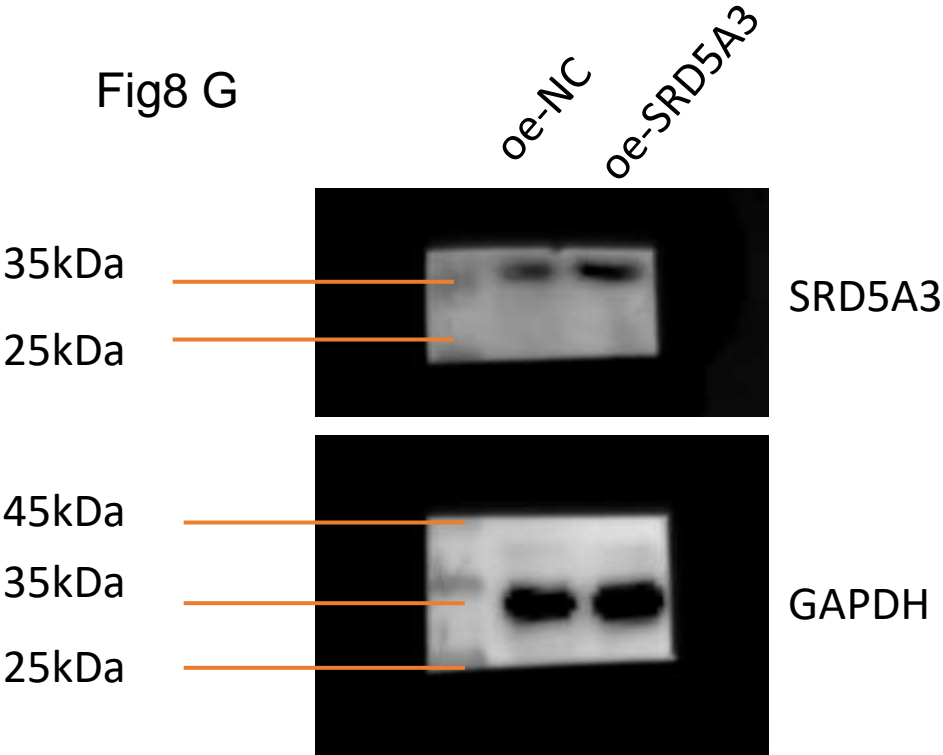

Fig11 A

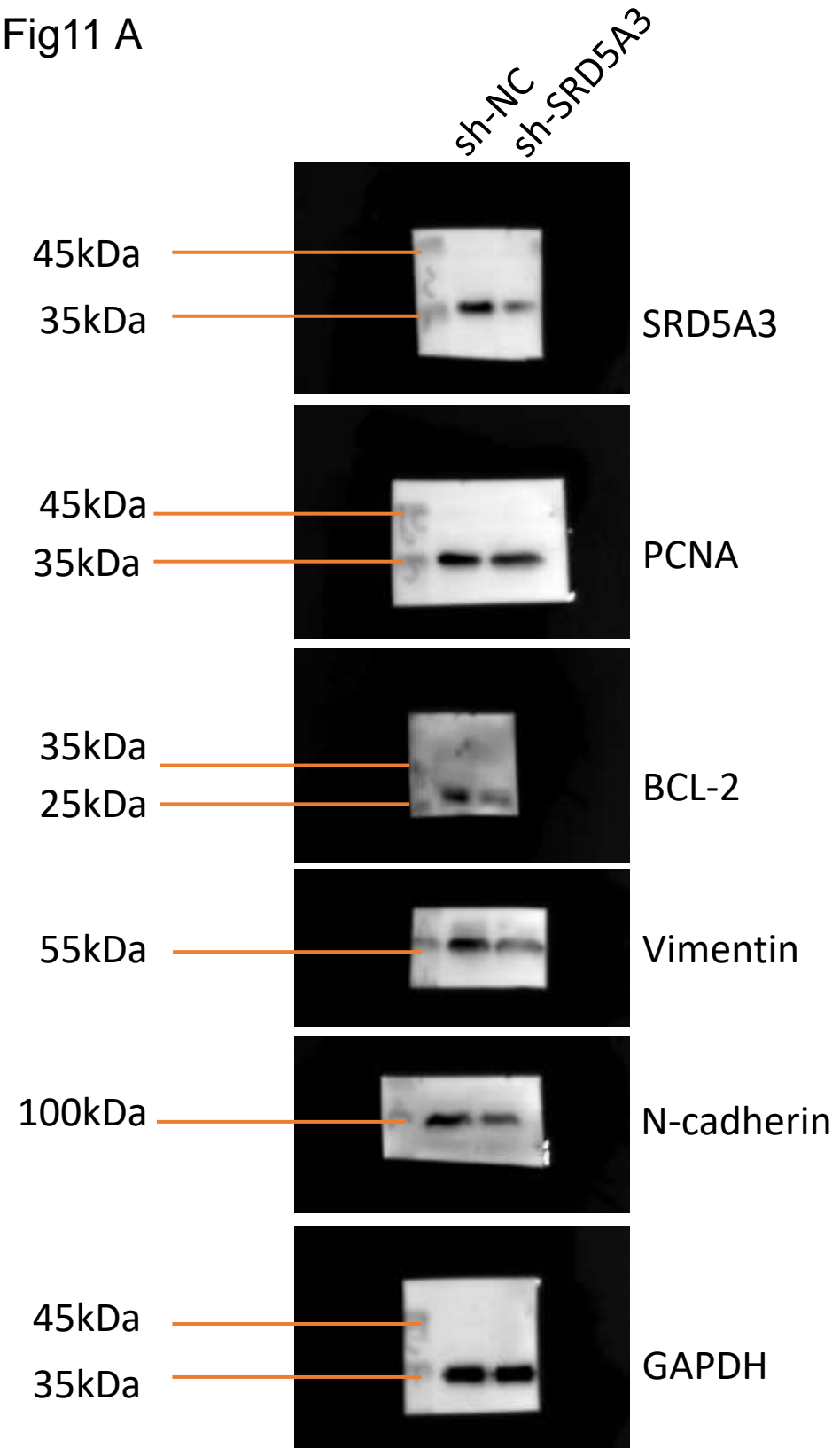

Fig11 B

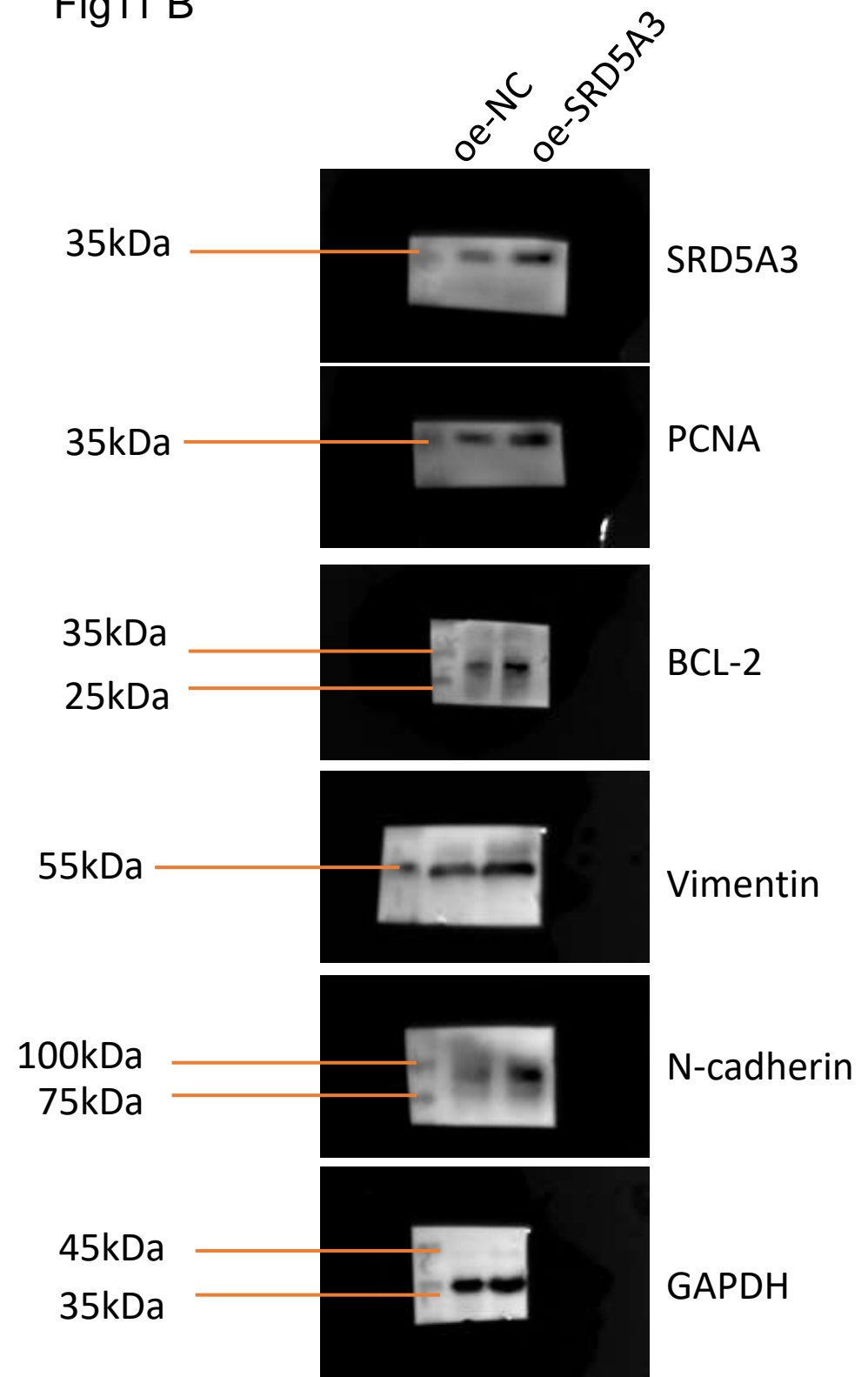

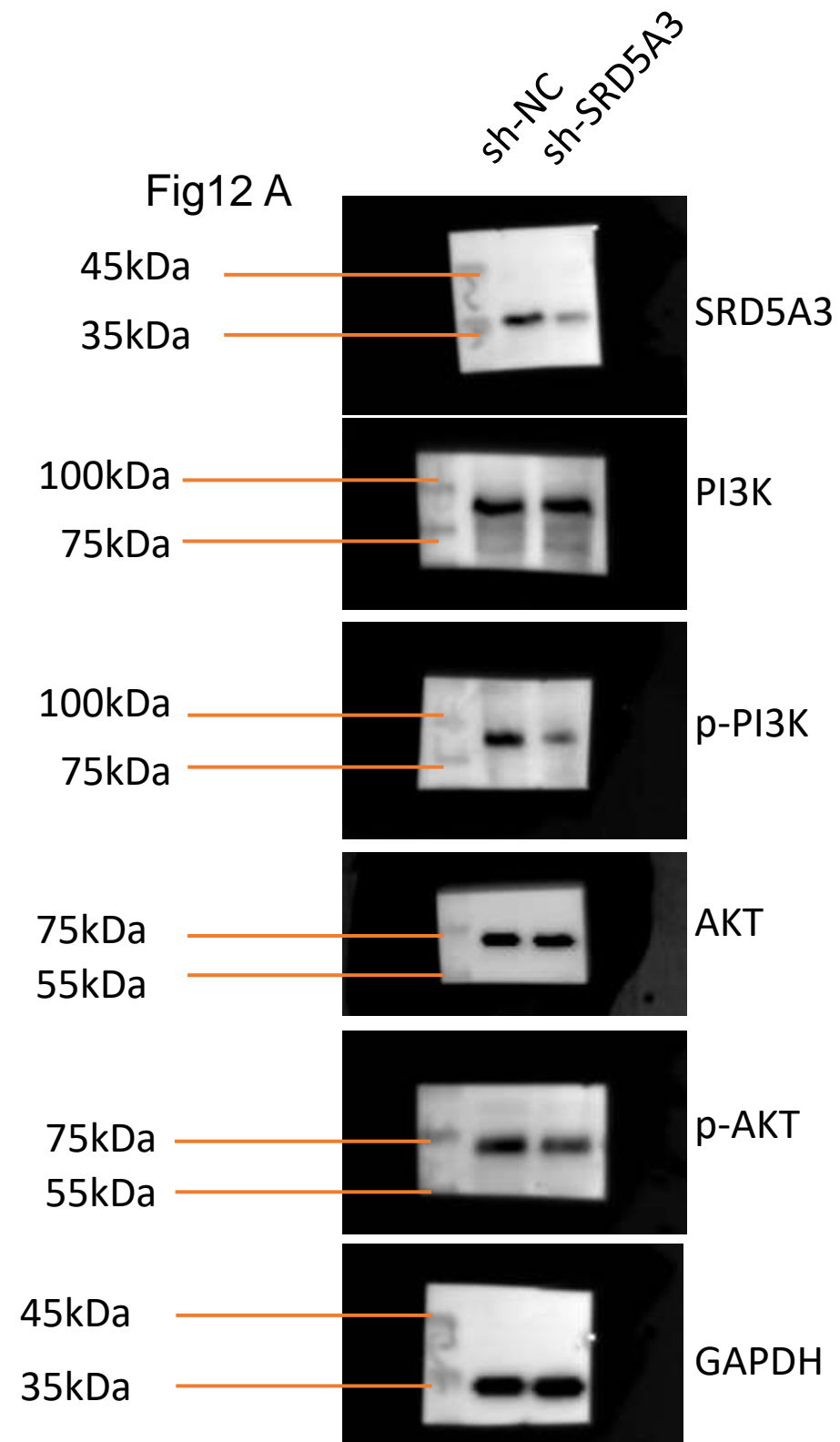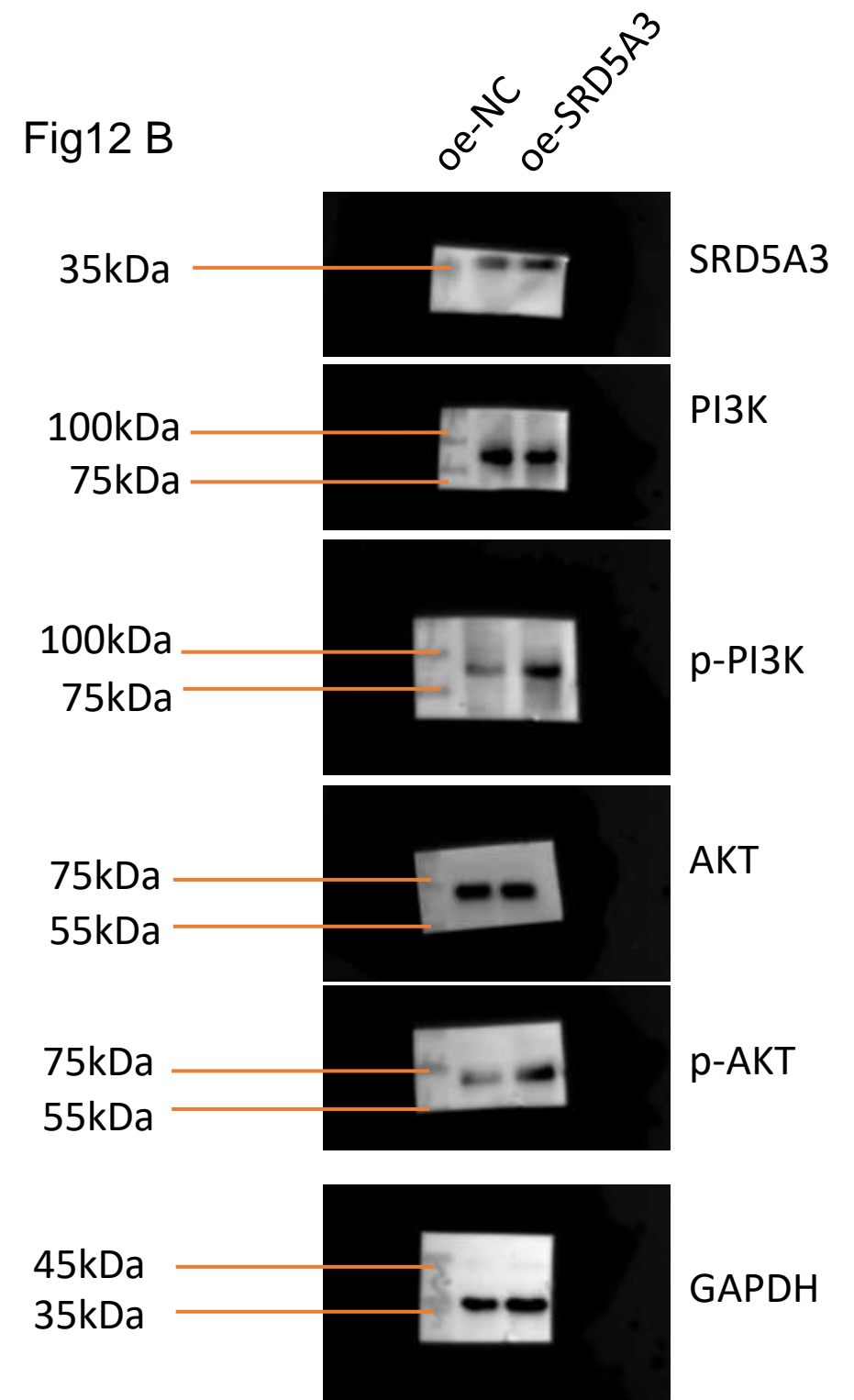

sh-NC  
sh-NC+3-MA  
sh-SRD5A3  
sh-SRD5A3+3-MA

Fig13 A

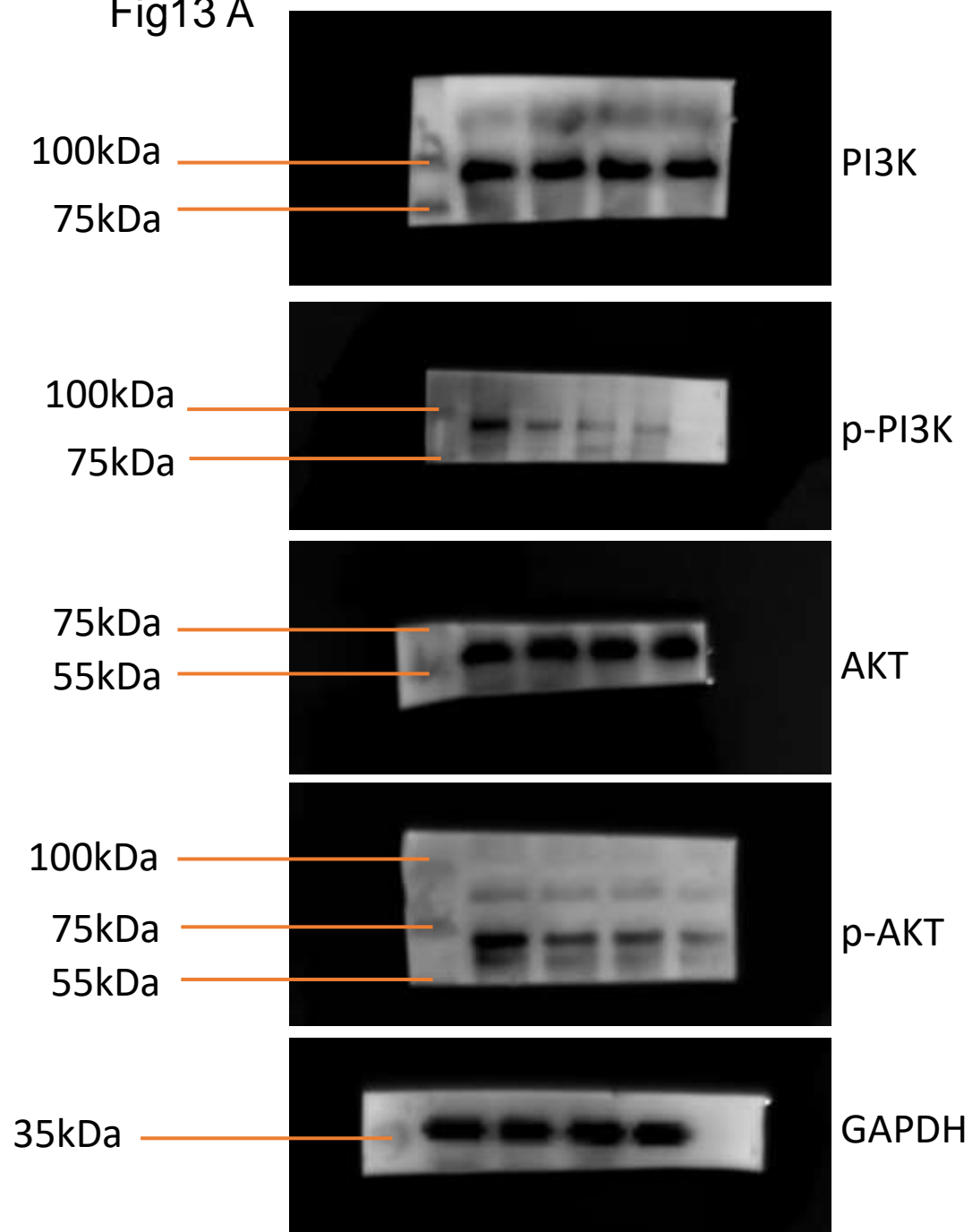

oe-NC  
oe-NC+3-MA  
oe-SRD5A3  
oe-SRD5A3+3-MA

Fig13 B

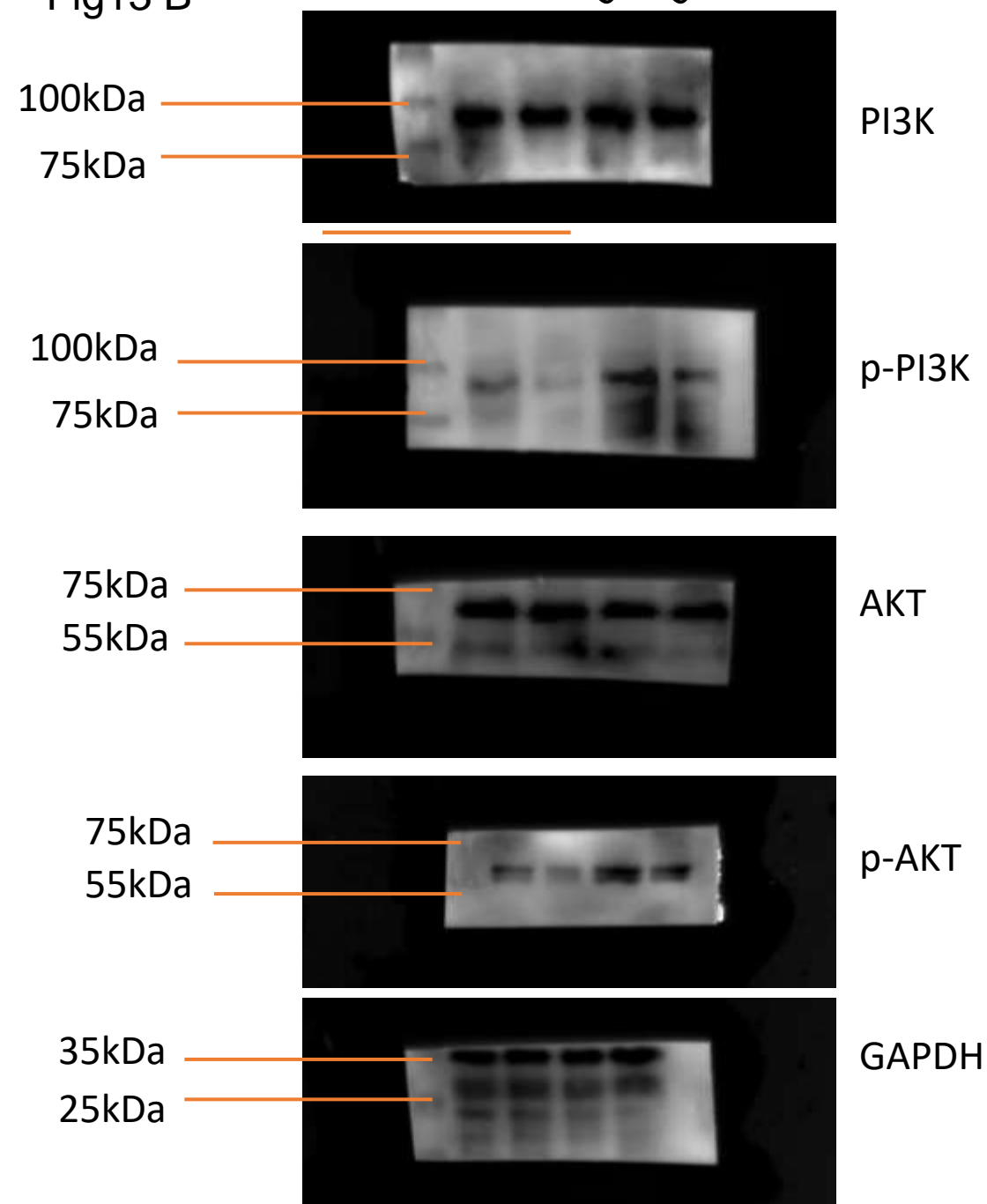

Supplement: S2 File — (PDF) [file pone.0323965.s002.pdf]
